# POTEE drives colorectal cancer development via regulating SPHK1/p65 signaling

**DOI:** 10.1038/s41419-019-2046-7

**Published:** 2019-11-13

**Authors:** Zhiyong Shen, Xiaochuang Feng, Yuan Fang, Yongsheng Li, Zhenkang Li, Yizhi Zhan, Mingdao Lin, Guoxin Li, Yi Ding, Haijun Deng

**Affiliations:** 1 Department of General Surgery, Nanfang Hospital, Southern Medical University, 1838 North Guangzhou Ave., 510515 Guangzhou, Guangdong Province China; 2 Department of Radiation Oncology, Nanfang Hospital, Southern Medical University, 1838 North Guangzhou Ave., 510515 Guangzhou, Guangdong Province China; 3 Department of Pathology, Nanfang Hospital, Southern Medical University, 1838 North Guangzhou Ave., 510515 Guangzhou, Guangdong Province China

**Keywords:** Colorectal cancer, Predictive markers

## Abstract

Aberrant gene expression plays critical roles in the development of colorectal cancer (CRC). Here we show that POTEE, which was identified as a member E of POTE ankyrin domain family, was significantly upregulated in colorectal tumors and predicted poor overall survival of CRC patients. In CRC cells, POTEE could act as an oncogene and could promote cell growth, cell-cycle progression, inhibit apoptosis, and elevates xenograft tumor growth. Mechanically, we used microarray analysis and identified a POTEE/SPHK1/p65 signaling axis, which affected the biological functions of CRC cells. Further evaluation showed that overexpression of POTEE could increase the protein expression of SPHK1, followed by promoting the phosphorylation and activation of p65 protein. Altogether, our findings suggested a POTEE/SPHK1/p65 signaling axis could promote colorectal tumorigenesis and POTEE might potentially serve as a novel biomarker for the diagnosis and an intervention of colorectal cancer.

## Introduction

Colorectal cancer is the third most commonly diagnosed cancer (10.2% of the total cases) and the second leading cause of cancer related deaths (9.2% of the total cancer deaths) in 2018 globally^1^. Both incidence and death rates of colorectal cancer are increasing rapidly and maintain an upward trend in Asian countries^2^. The global burden of colorectal cancer (CRC) is expected to increase by 60% to more than 2.2 million new cases and 1.1 million deaths by 2030^2–4^. Exploring related genes in the development of CRC and finding important links that affect the biological characteristics of CRC are critical ways to understand the malignancy of tumors and to improve the survival and prognosis of CRC patients^5^.

POTE (Prostate, Ovary, Testes, and Embryo) is a newly detected gene family that contains ankyrin and spectrin domains and express in a variety of human cancers^6–8^. This family has 11 exons and 10 introns and spans 32 kb of chromosome 21q11.2 region, which consists of at least 10 highly homologous genes located on chromosomes 2, 8, 13, 14, 15, 18, 21, and 22^7^. POTEE is a paralogs located at chromosome 2 that contains three distinct regions: N-terminal cysteine-rich domains followed by seven ankyrin repeats and C-terminal spectrin-like helices^9,10^. Previous studies have shown that POTEE was only weakly expressed in normal tissues of prostate and breast, but its expression was significantly elevated in their tumor counterparts^8,11^. It was also reported that serum POTEE level in non-small cell lung cancer (NSCLC) patients was associated with advanced TNM stage and might serve as a potential prognostic indicator of NSCLC patients^12^. In addition, upregulation of POTEE also indicated poorer prognosis of ovarian cancer patients^13^. Recently, a study showed that overexpression of POTEE in macrophages and its subtype could provide a platform for mTOR and Rictor binding thereby resulting in activation of mTORC2^10^. Although above-mentioned studies suggested a potential oncogenic role of POTEE in various cancer types, its biological functions and tumorigenesis mechanisms remains largely unknown. In colorectal cancer, the dysregulation of POTEE are still undefined to our knowledge.

Here, we conducted researches on detecting the expression status and clinical characteristics of POTEE in colorectal cancer tumor samples and cells, with the aim to elucidate the oncogenic roles and potential mechanisms of POTEE both in vitro and in vivo. Our study provides new mechanistic insights into the roles of POTEE in promoting SPHK1/p65 signaling, which might server as a potential biomarker and a novel intervention target for colorectal neoplasia.

## Results

### POTEE is upregulated and predicts poor clinical outcome in CRC patients

**Fig. 1:**
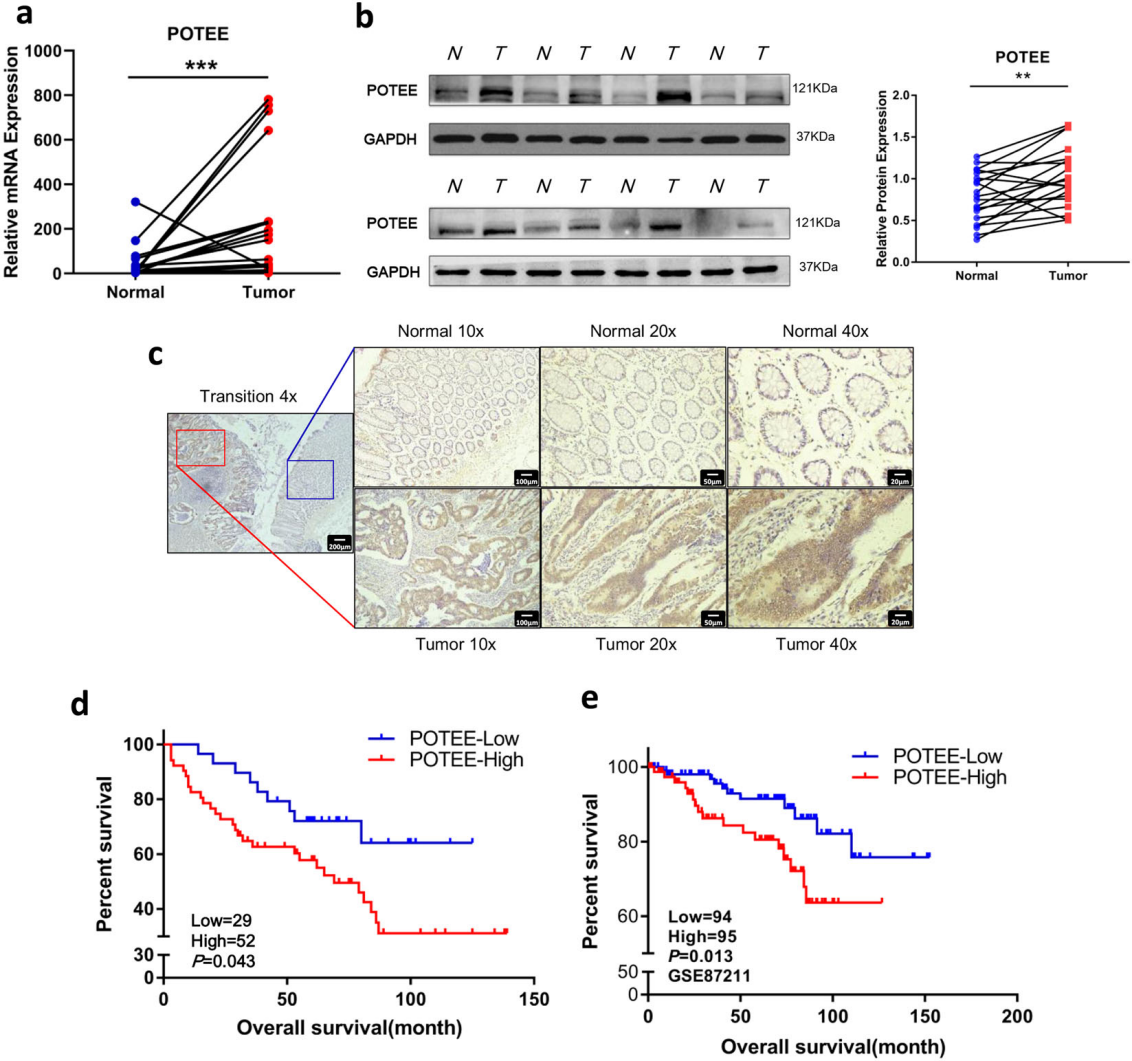
POTEE is upregulated and predicts poor clinical outcome in CRC patients. **a** The relative mRNA levels of POTEE in 20 paired CRC and adjacent normal tissues. Results are shown as mean ± SD. *P < .05, **P < .01, ***P < .001, based on Paired *t*-test. **b** Western blots of POTEE protein in 20 pairs of CRC tissues, GAPDH was loaded as a control. Relative protein expression were measured by Image J and shown as mean ± SD. *P < .05, **P < .01, ***P < .001, based on Paired *t*-test. **c** Representative staining of POTEE with immunohistochemistry (IHC) analysis. **d** Overall survival rates of the CRC patients (*n* = 81) grouped by POTEE protein expression levels. **e** Overall survival of patients with CRC stratified by POTEE mRNA expression from GSE87211 dataset

To explore the expression of POTEE in CRC, we firstly carried out quantitative real-time polymerase chain reaction (qRT-PCR) to analyze the messenger RNA (mRNA) expression of POTEE in 20 pairs of CRC samples and their normal counterparts. The results showed that POTEE was significantly upregulated in tumors (19/20, 95%) in comparison with their paired normal mucosa (Fig. 1a). Consistent with mRNA level, the protein expression analyzed by western blot and immunohistochemistry (IHC) also verified the elevated expression of POTEE in colorectal tumor tissues (Fig. 1b, c). What’s more, ours results further revealed the intense nuclear and weak cytoplasmic staining of POTEE in the epithelial component of carcinomas (Fig. 1c; Supplementary Fig. 1a. b).

Next, we went on investigating the clinical relevance of POTEE expression with CRC patients and finally 81 samples with visible POTEE staining by IHC and complete follow-up data from our center were included for our following analysis. We detected strong POTEE expression in almost 65% of colorectal tumor samples examined, and most of adjacent normal mucosa showed weak or absent POTEE signal. Kaplan–Meier survival analysis showed high POTEE expression predicted poorer overall survival (OS) than those with weak POTEE expression (Fig. 1d). Clinicopathological characteristics of 81 CRC patients showed that high POTEE expression was obviously associated with more lymph nodal metastasis, higher clinical stage, and larger tumor size (Table 1). External dataset also validated that high POTEE mRNA level could also predicted significantly poorer overall survival in GSE87211 with 189 rectal cancer patients involved (Fig. 1e). Together, these data indicated that POTEE is upregulated in human CRC tissues and overexpression of POTEE correlates with malignant phenotypes as well as unfavorable prognosis of colorectal cancer.

**Table 1:** Correlation between expression of POTEE and clinical characteristics of CRC patients

Characteristics	Cases	POTEE Immunostaining		χ^2^ value	*P*
		Decreased expression	High expression		
Gender				0.200	0.655
Male	42	16 (38.1%)	26 (61.9%)		
Female	39	13 (33.3%)	26 (66.7%)		
Age (years)				1.454	0.228
<60	32	14 (43.8%)	18 (56.3%)		
≥60	49	15 (30.6%)	34 (59.4%)		
Lymph nodal metastasis				4.464	0.029
N0	49	22 (44.8%)	27 (45.2%)		
N1/N2	32	7 (21.8%)	25 (78.1%)		
TNM				5.901	0.013
I/II	47	22 (46.8%)	25 (53.2%)		
III/IV	34	7 (20.6%)	27 (79.4%)		
Tumor size^a^				4.500	0.042
<5 cm	46	21 (45.7%)	25 (54.3%)		
≥5 cm	34	8 (23.5%)	26 (76.5%)		

^a^1cases missing Tumor size data

### POTEE plays oncogenic roles in CRC cells

**Fig. 2:**
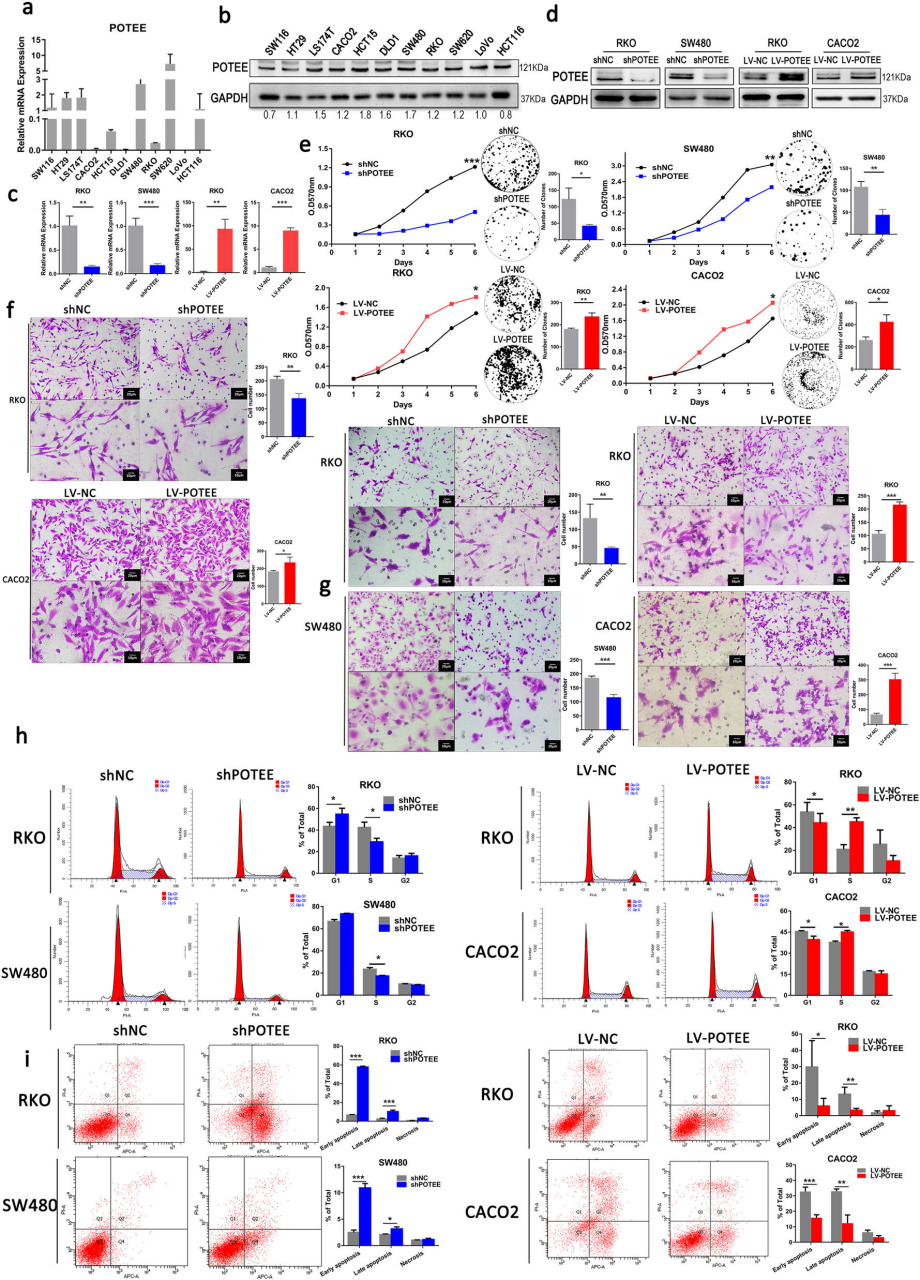
POTEE enhances tumorigenesis and inhibits apoptosis of CRC cells. **a**, **b** qRT-PCR (**a**) and western blot (**b**) were performed to examine POTEE endogenous expression in 11 colon cancer cell lines. **c**, **d** Transfection efficiency was detected by qRT-PCR (**c**) and western blot (**d**). Results are shown as mean ± SD (*n* = 3). ^*^P < .05, ^**^P < .01, ^***^P < .001 based on Student *t*-test. **e** MTT (left) and colony formation assays (right) in indicated cell lines with different POTEE expression. Statistics were measured by 2-way ANOVA or Student *t*, ^*^P < .05, ^**^P < .01, ^***^P < .001. **f**, **g** Migration (**f**) and invasion (**g**) assay in indicated cell lines with different POTEE expression. Statistics were measured by Student t, ^*^P < .05, ^**^P < .01, ^***^P < .001. **h** Cell-cycle distribution analysis. Results are shown as mean ± SD (*n* = 3). ^*^P < .05, ^**^P < .01, ^***^P *<* .001 based on Student *t*-test. **i** Apoptosis analysis with annexin V-APC/PI staining. Cells were exposed to 50 µg/ml 5-Fluorouracil for 36–48 h. Results are shown as mean ± SD (*n* = 3). ^*^P < .05, ^**^P < .01, ^***^P *<* .001 based on Student *t*-test

Given the significant expression difference and clinical relevance of POTEE, we further evaluated the functional roles of POTEE in CRC cells. We firstly examined endogenous expression of POTEE in 11 colorectal cancer cell lines by qRT-PCR and western blot analysis (Fig. 2a, b). Then, POTEE-targeting shRNA (shPOTEE) or corresponding controls (shNC) were used to establish stable POTEE-knockdown cell lines in SW480 and RKO cells with relatively high and moderate endogenous POTEE expression, respectively. While GFP-tagged POTEE-expressing recombinant lentivirus (LV-POTEE) or control vectors (LV-NC) were introduced into RKO and CACO2 cells. The transfection efficiency was verified by qRT-PCR and western blot analysis (Fig. 2c, d). Results showed that POTEE knockdown in RKO and SW480 cells significantly inhibited cell proliferation and colony formation, while ectopic expression of POTEE in RKO and CACO2 cells significantly promoted cell growth (Fig. 2e). In addition, wound-healing assays and transwell migratory and invasive assays revealed that upregulated POTEE could remarkably enhanced the migratory and invasive ability of RKO and CACO2 cell lines, while knockdown POTEE in RKO and SW480 exerted opposite effects and weaken the metastasis of cells. (Fig. 2f, g; Supplementary Fig. 2a, b). As cell proliferation, cell-cycle regulation and apoptosis are tightly bound^14,15^, so we next evaluated the effect of POTEE on cell-cycle regulation in cells with different POTEE status. Results showed POTEE overexpression resulted in decreased cell proportions in the G1 phase while significantly elevated cell fractions in the S phase in RKO and CACO2 cells, but knockdown of POTEE reversed cell-cycle progression in SW480 and RKO cells (Fig. 2h). Moreover, to further identify whether POTEE could play a role in apoptosis control, we treated cells with 5-Fluorouracil (5-Fu) to induce cell apoptosis, results revealed that knockdown of POTEE led to a sharp increase of apoptosis as compared with their negative controls. However, apoptosis induced by 5-Fu treatment was significantly rescued after POTEE overexpression (Fig. 2i). Collectively, these data supported the oncogenic role of POTEE in inducing proliferation, migration and invasion, inhibiting apoptosis and promoting cell-cycle progression in CRC cells in vitro.

### RNA-Seq reveals POTEE-regulated signaling pathways in CRC cells

**Fig. 3:**
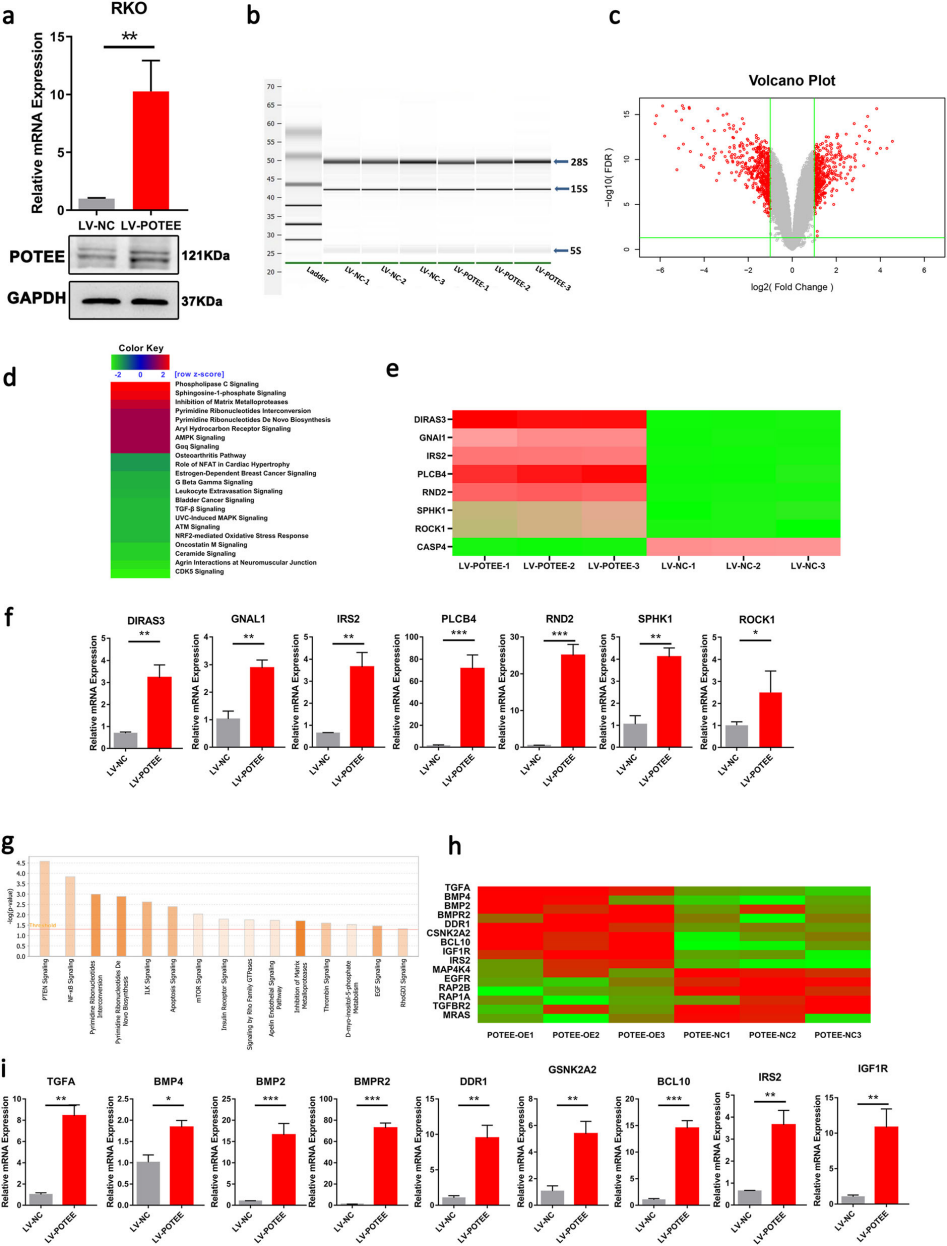
RNA-seq reveals POTEE-regulated signaling pathways in CRC. **a** qRT-PCR (left) and western blot (right) showing POTEE expression in RKO cells with POTEE overexpression or mock cells. Results are shown as mean ± SD (*n* = 3). ^*^P < .05, ^**^P < .01, ^***^P *<* .001 based on Student *t*-test. **b** The total mRNA integrity was tested on the Agilent 2100 bioanalyzer used the Eukaryote Total RNA Nano assay. **c** Differentially expressed genes (2-fold) between mock and LV-POTEE RKO cells were determined by RNA-Seq and shown by volcano plot. **d** IPA z-score analysis. IPA z-score > 2 or < -2 was considered to be significant activation or inhibition. Analysis result showed that the IPA z-score of S1P signaling was 2.449. **e** Heat map showing the microarray result for the differentially expression genes involved in S1P pathway. Scale bar: red (upregulated), green (downregulated). **f** qRT-PCR was performed to examine seven upregulated genes involved in S1P molecules network in RKO cells with POTEE overexpression or mock cells. Results are shown as mean ± SD (*n* = 3). ^*^P < .05, ^**^P < .01, ^***^P *<* .001 based on Student *t*-test. **g** Histogram sort based on *P*-value, and the horizontal line represented actively classic pathway. **h** Heat map showing the microarray result for the differentially expression genes involved in NF-κB pathway. Scale bar: red (upregulated), green (downregulated). **i** Indicated upregulated 9 genes involved in NF-κB pathway measured by qRT-PCR. Results are shown as mean ± SD (*n* = 3). ^*^P < .05, ^**^P < .01, ^***^P *<* .001 based on Student *t*-test

To better detect POTEE-mediated molecular pathways in CRC cells, we conducted a genome-wide analysis to globally characterize POTEE-regulated transcriptome changes. Total RNAs from RKO cells with LV-NC or LV-POTEE were subjected to transcriptomic sequencing (RNA-Seq). The expressions of POTEE mRNA and protein were validated prior to RNA-Seq (Fig. 3a). After assessing the intactness of the samples and verifying rare degraded mRNA (Fig. 3b), microarray analysis was performed and a total of 638 genes were identified to be significantly regulated in POTEE-overexpressed cells with respect to control cells (Fig. 3c; Supplementary Fig. 3). Subsequent Ingenuity Pathway Analysis (IPA) of the significantly regulated genes revealed that the top-three ranked molecular pathways based on IPA z-score included “Phospholipase C”, “Sphingosine-1-phosphate (S1P)”, and “Inhibition of Matrix Metalloproteases”. In the meantime, when analyzed according to different *P*-value, the most enriched pathways were “PTEN Signaling”, “NF-κB Signaling”, and “Pyrimidine Ribonucleotides Interconversion” in sequence (Fig. 3d, g).

According to the results of microarray analysis, genes involved in S1P molecules network was most significantly enriched and we identified seven genes (DIRAS3, GNAL1, IRS2, PLBC4, RND2, SPHK1, ROCK1, fold change > 1.5) were sharply upregulated and one gene (CASP4, fold change > 1.5) was shown to be downregulated (Fig. 3e) in S1P signaling and.the seven upregulated genes were chosen for further validation with qRT-PCR (Fig. 3f). As was reported, intracellular activation of S1P signaling was catalyzed by the key and rate-limiting sphingosine kinase isoform, SPHK1^16^, which was also found to be significantly increased in POTEE-overexpressed RKO cells and was focused in our following detection.

As NF-κB pathway was also the top activated POTEE-mediated carcinogenic pathways ranked by *P*-value (Fig. 3g), and it was reported that NF-κB pathway was a main downstream of S1P signaling^17–19^, so we further did heat map according to the nine genes significantly upregulated and five downregulated genes involved in NF-κB pathway, and qRT-PCR analysis was also undertook to identify the accuracy of the microarray sequence (Fig. 3h, i).

### POTEE facilitates proliferation through upregulating SPHK1

**Fig. 4:**
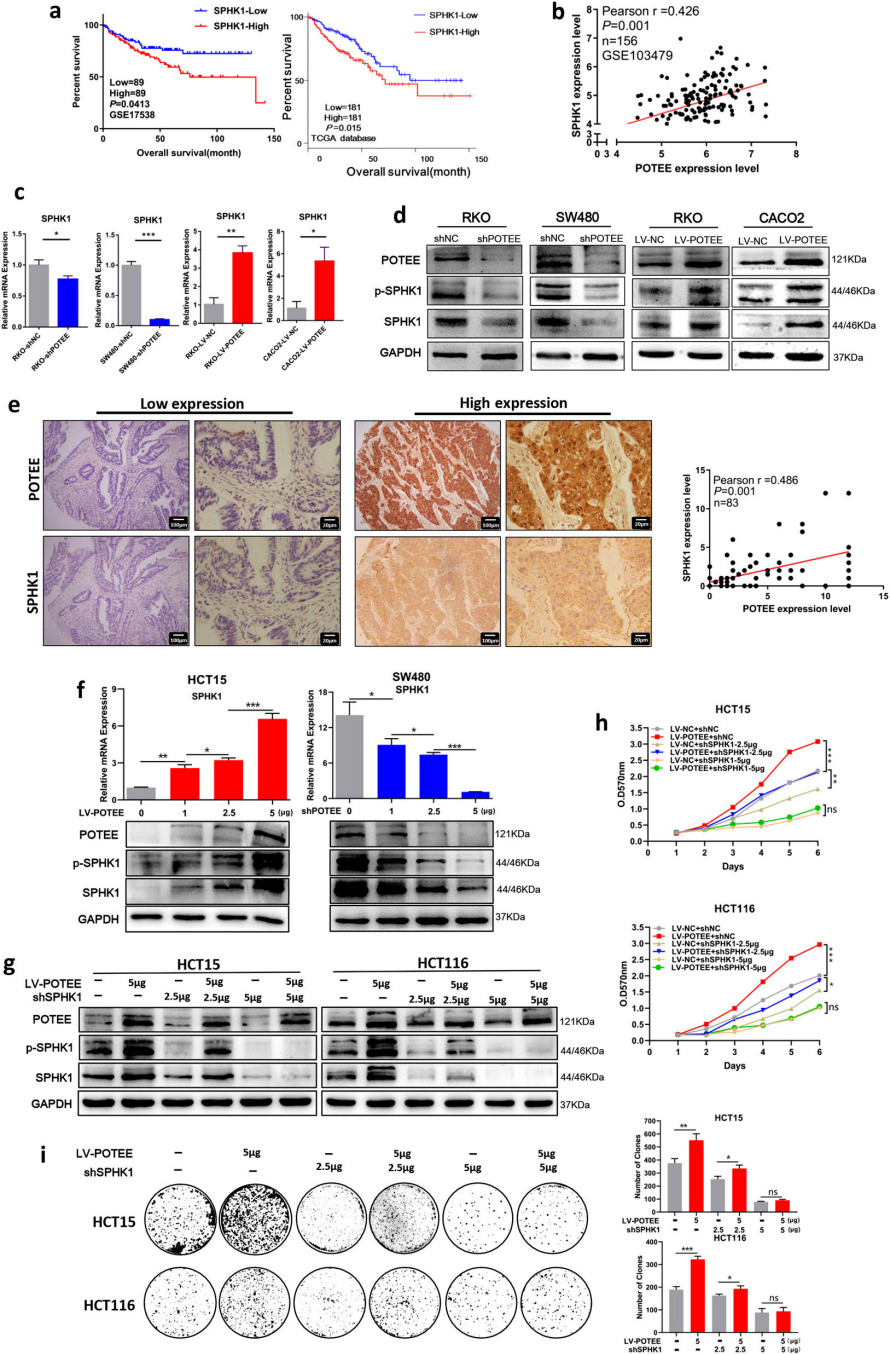
POTEE facilitates proliferation through up-regulating SPHK1. **a** Kaplan–Meier survival curves depicting overall survival of patients with CRC grouped by SPHK1 mRNA expression levels. Data were deposited to GSE17538 dataset (left) and TCGA database (right). **b** Correlation between mRNA expression of POTEE and SPHK1 based on Pearson χ2 test, data was deposited to GSE103479 dataset. **c** qRT-PCR was performed to detect mRNA level of SPHK1 in indicated cell lines. Results are shown as mean ± SD (*n* = 3). ^*^P < .05, ^**^P < .01, ^***^P *<* .001 based on Student *t*-test. **d** Total and phosphorylated levels of SPHK1 (p-SPHK1) were analyzed by western blotting in the cell lines with different POTEE expression. **e**. Identification of the correlation between protein levels of POTEE and SPHK1 in CRC tissues with IHC analysis. Statistics were measured by Pearson χ^2^ test. **f** qRT-PCR (up) and western blot (down) showing POTEE and SPHK1 expression in HCT15 and SW480 with different doses of overexpression and knockdown in POTEE. Results are shown as mean ± SD (*n* = 3). ^*^P < .05, ^**^P < .01, ^***^P *<* .001 based on Student *t*-test. **g** Western blot detected indicated proteins with different doses of knockdown in POTEE. **h**, **i** MTT (**h**) and colony formation assays (**i**) in cells with different POTEE and SPHK1 expression. Statistics were measured by 2-way ANOVA or Student *t*, ^*^P < .05, ^**^P < .01, ^***^P < .001

As SPHK1 is the key sphingosine kinase that drives S1P signaling^20,21^, we then went on detecting the possible involvement of SPHK1 in POTEE-mediated molecular downstream. It’s reported that high expression level of SPHK1 promoted the development of tumor malignancy and significantly correlated with occurrence and poor prognosis of cancer patients^16,20^, so we firstly analyzed the correlation between SPHK1 expression and the survival of CRC patients. Results showed that increased SPHK1 mRNA level could predict poorer overall survival than those patients with relatively low SPHK1 expression in GSE17538 dataset with 178 CRC samples and in TCGA database with 362 colorectal cancer patients involved (Fig. 4a). We then analyzed the correlation of POTEE and SPHK1 in GSE103479 dataset, and results revealed that the mRNA expression of POTEE and SPHK1 were positively correlated (r = 0.426, *P* = 0.001) (Fig. 4b). To further verify SPHK1 expression under different POTEE status, we performed qRT-PCR and western blot analysis and found that both the mRNA and protein level of SPHK1 were significantly upregulated in CACO2 and RKO cells with POTEE-overexpression, while knockdown of POTEE repressed SPHK1 expression (Fig. 4c, d), and the protein level of phosphorylated SPHK1 showed the same change tendency according to different POTEE expression (Fig. 4d). In addition, we performed IHC staining with both anti-POTEE and anti-SPHK1 antibodies on our tissue microarrays with 83 CRC patients involved to measure the correlation between them, and results also revealed a significantly positive correlation between POTEE and SPHK1 on protein level (Fig. 4e).

In order to further determine the role of SPHK1 in the regulation of POTEE-mediated oncogenesis, we firstly detected endogenous expression of SPHK1 in 11 colorectal cancer cell lines by qRT-PCR and western blot analysis to analyze the endogenous consistency between POTEE and SPHK1, and results showed that the endogenous expression level of SPHK1 was finely accordant with POTEE in quite a part of these cell lines, and we additionally chose HCT15 and HCT116 cells in our following experiments for their moderate expression of both POTEE and SPHK1 (Supplementary Fig. 4a, b). To explore the role of SPHK1 in POTEE-mediated tumor promoting functions, we firstly transfected gradient increased doses of POTEE-expressing recombinant lentivirus or POTEE-targeting shRNA into HCT15 and SW480 cells, respectively. Results showed that gradient overexpression or knockdown of POTEE led to a progressive upregulation or loss of downstream SPHK1 expression, which firmly indicated the key role of POTEE in regulating SPHK1 in CRC cells (Fig. 4f). Then, we used POTEE-overexpressed cells to generate cells with different levels of SPHK1 knockdown, and results revealed that different doses of knockdown in SPHK1 could progressively block POTEE-mediated SPHK1 elevation (Fig. 4g; Supplementary Fig. 4c). To better elucidate the functional changes, we conducted MTT tests and colony formation assays to determine whether suppressed SPHK1 could reverse POTEE-mediated cell proliferation. Results revealed that low and moderate inhibition of SPHK1 was capable enough to partly rescue the proliferative ability of indicated cell lines, and CRC cells with POTEE overexpression was more sensitive to SPHK1 knockdown for significantly severer inhibition of cell viability than their negative controls, and the cell growth was extremely suppressed after extensive SPHK1 knockdown (Fig. 4h, i; Supplementary Fig. 4d, e). Hence, our results suggested SPHK1 was an oncogene and was indispensable for POTEE-mediated oncogenic functions in CRC cells.

### p65 plays as a functional downstream molecular of POTEE/SPHK1 axis

**Fig. 5:**
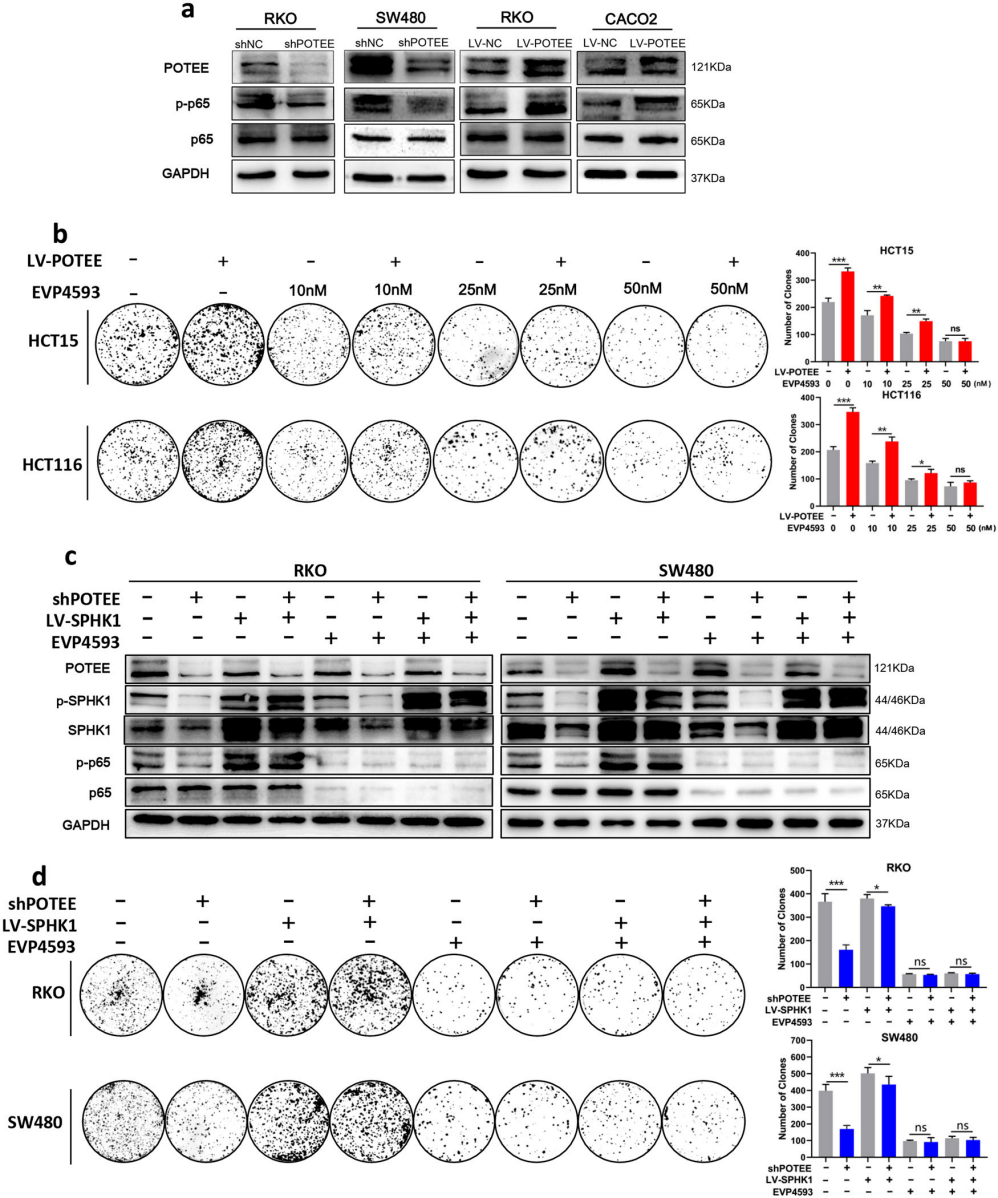
p65 plays as a functional downstream molecular of POTEE/SPHK1 axis. **a** Total and phosphorylated levels of p65 (p-p65) were analyzed by western blotting in the cell lines with POTEE knockdown or POTEE overexpression. **b** Colony formation assays with indicated concentration of EVP4593 or DMSO. Statistics were measured by Student *t*, ^*^P < .05, ^**^P < .01, ^***^P < .001. **c** Western blot analysis in indicated cell lines with EVP4593 (0 nM, 10 nM, 25 nM, 50 nM, 48 h) or DMSO treatment. **d** Colony formation assays with indicated treatment. Statistics were measured by Student *t*, ^*^P < .05, ^**^P < .01, ^***^P < .001

As previous studies have verified that SPHK1-mediated carcinogenesis via the activation of NF-κB pathway, and in light of our microarray results earlier (Fig. 3b), we went on detecting the activation of NF-κB signaling in CRC cells with different POTEE overexpression. Due to the expression of p65 and phosphoinositide-p65 (p-p65) are key mediators of NF-κB activation^22,23^, we firstly analyzed their expressions and found that although there showed no significant difference in p65 protein expression, the phosphorylated levels of p65 (p-p65) decreased in POTEE-knockdown cells and increased in POTEE-overexpressed cells (Fig. 5a).

We then tested whether NF-κB activation was required for POTEE-mediated proliferation of CRC cells. We used a pharmacologic approach to inhibit the activation of NF-κB with EVP4593 (Selleck Chemicals, America), which targeted p65^24,25^ at different concentration. Results showed EVP4593 could suppress POTEE-mediated cell proliferation in a dose-dependent manner, and although EVP4593 could inhibit the cells growth of both control and POTEE-overexpressed cells, stronger repression was seen in cells with POTEE-overexpression, similarly, high concentration of EVP4593 could completely blocked cell growth induced by POTEE overexpression (Fig. 5b; Supplementary Fig. 5b, c, d). Additionally, we used RKO and SW480 with POTEE knockdown to overexpress SPHK1 and found that SPHK1 overexpression could rescue the protein level of p-p65 in cell lines with POTEE knockdown, but high concentration of EVP4593 treatment could inhibited this upregulation. Likewise, knockdown SPHK1 was also capable of reducing p-p65 levels in POTEE-overexpressed cell lines (Fig. 5c; Supplementary Fig. 5e). What’s more, the results of colony formation assays suggested that gradient inhibition of p-p65 could progressively suppress cell proliferation promoted by POTEE/SPHK1 axis, and excessive inhibition eliminated the difference of cell number affected either by POTEE or SPHK1 (Fig. 5d). Collectively, these results implied that POTEE contributed to the proliferation of CRC cells through the SPHK1-NF-κB signaling axis.

### Knockdown POTEE could decrease tumor growth in vivo

**Fig. 6:**
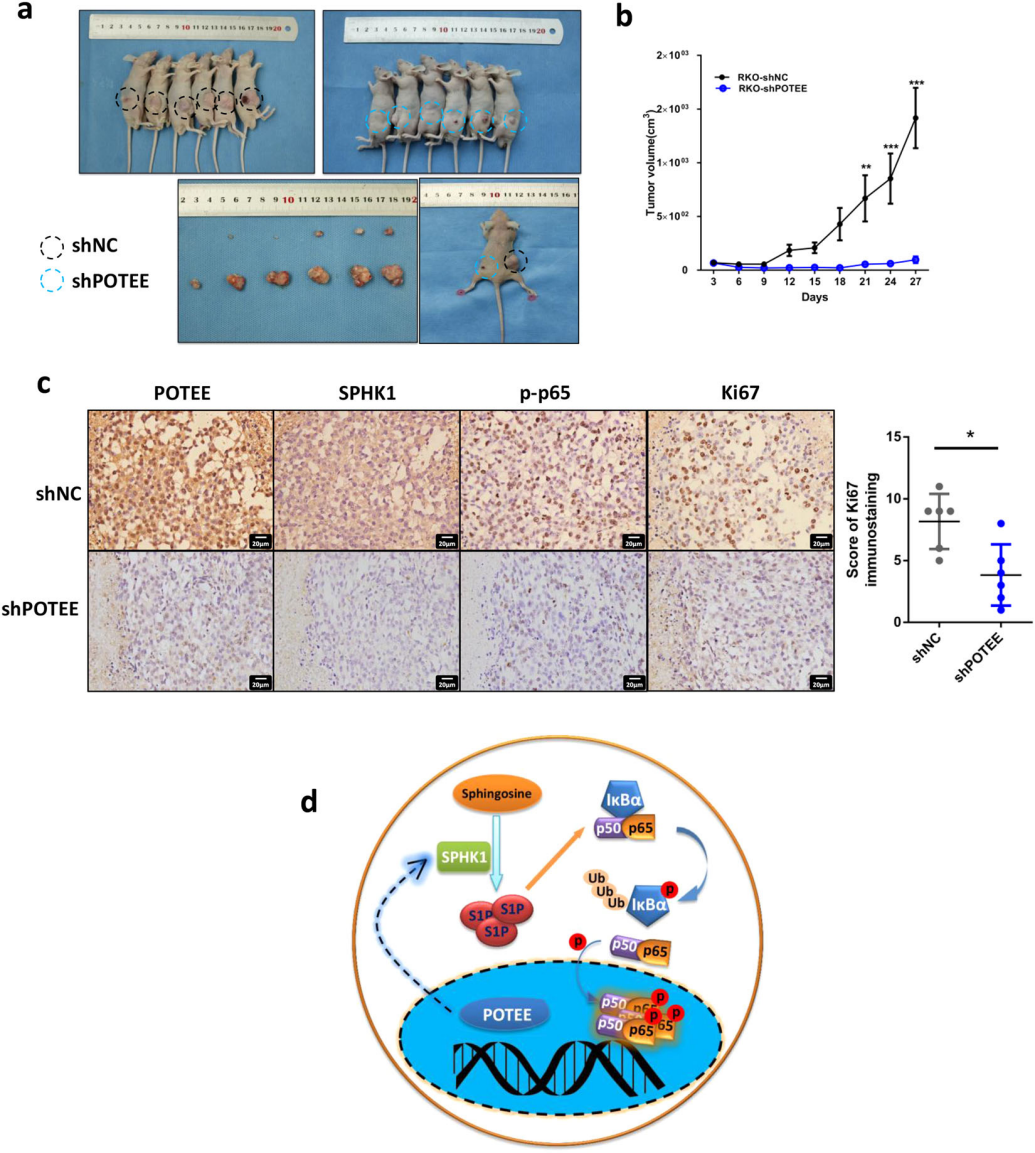
Knockdown POTEE could decrease tumor growth in vivo. **a** Representative photographs showing the tumors in mice after the subcutaneous transplantation of RKO cells with POTEE knockdown or control cells. **b** Tumor growth curves measured at the indicated time points between POTEE knockdown and control groups. Statistics were measured by 2-way ANOVA, ^*^P < .05, ^**^P < .01, ^***^P < .001. **c** Representative IHC photographs showed positive relationship between POTEE, SPHK1, p-p65, Ki67 expression in tumor tissues (left). Results of Ki67 immunohistochemistry staining were evaluated by the staining scores (right). Results are shown as mean ± SD (*n* = 6). ^*^P < .05 based on Paired *t*-test. **d**. Schematic depiction of the mechanisms underlying CRC proliferation via POTEE/SPHK1/p65 signaling

Considering our in vitro findings, we further tested the functional roles of POTEE in vivo with subcutaneous xenograft models. Results revealed that RKO cells with POTEE-knockdown suppressed tumor growth by 93.2% when compared to their negative control cells (Fig. 6a, b). POTEE silence significantly inhibited cell proliferation in the tumor sections as determined by Ki67 staining (Fig. 6c). Furthermore, knockdown of POTEE also led to decreased expression of SPHK1 and p-p65 in the tumors (Fig. 6c). These findings highlighted an essential role of POTEE/SPHK1/p65 signaling in promoting colorectal tumor growth (Fig. 6d).

## Discussion

POTE is a gene family and several studies have demonstrated that POTE genes were associated with a variety of human cancers^8^. POTEE was firstly detected in prostate cancer and its aberrant expressions was then identified in other cancer types^6,7^. Based on omics-based whole-genome transcriptome and whole-proteomic profiling, researchers observed that POTEE was closely related to breast cancer and might be involved in disease progression^11^. In Tripathi’s research, POTEE could interact with mTOR and Rictor (Rapamycin-insensitive companion of mTOR) proteins in tumor associated macrophages of prostate cancer, and then enhanced cell growth and proliferation through immune modulation^10^. Furthermore, recent findings indicated the roles of POTEF, one of homologous protein of POTEE, in the regulation of apoptosis in prostate cancer cells^26^. Despite the possible oncogenic roles of POTEE in different cancer types, its expression pattern, functions, mechanism largely remain obscure and need further study.

In this work, we conducted a more detailed investigation of the expression of POTEE and the relationships among POTEE expression and the prognosis and clinicopathological parameters of CRC patients. Specifically, the mRNA and protein expression of POTEE were significantly upregulated in human CRC tumor samples and cell lines. High POTEE level was positively associated with poor outcomes and malignant phenotypes of CRC patients. Our gain-of-function and loss-of-function experiments in vitro and in vivo clearly suggested a oncogenic role of POTEE in regulation of cell proliferation, migration and invasion, cell-cycle progression and apoptosis repression from both in vitro and in vivo evidence.

Since the molecular pathways associated with POTEE still remained obscure, we performed an mRNA microarray between POTEE-overexpressed RKO cells and control cells, and Sphingosine-1-phosphate (S1P) signaling was one of the most enriched pathways. S1P signaling is widely involved in many diseases, like inflammatory diseases and cancer progression, for its important and fundamental regulations of cell growth, apoptosis, and cell migration^21,27,28^. S1P is a bioactive type of sphingolipid, which is converted from ceramide and intracellular S1P was catalyzed mainly by the key sphingosine kinase isoforms, SPHK1^29^. Previous studies have demonstrated that high expression of SPHK1 in tumors was associated with poor prognosis of pulmonary, gastric, pancreatic and colonic cancer patients^30–33^ and high expression of phosphorylated SPHK1 could promote cell growth and metastasis^34^. In our work, we consistent expression of SPHK1 with POTEE from both endogenous and exogenous evidence, and they are also positively correlated in the tumor samples of CRC patients. On functional rescue experiments, we further identified SPHK1 was an indispensable downstream of POTEE-mediated cell growth. However, the mechanism for transcription induction between POTEE and SPHK1 remain unknown. As some research suggested that loss of SPHK1 was critical to p53-dependent tumor suppression mechanism, and SPHK1 inhibition combined with p53 overexpression played an important role in inducing cell death^35,36^; however, in our work, the expression level of p53 seemed to be little affected by different POTEE expression (Supplementary Fig. 5a), which indicated POTEE-mediated SPHK1 changes was independent of p53 status.

NF-κB signaling pathway contains five structurally related proteins, which form homodimers and heterodimers to upregulate or suppress genes expression by binding to κB elements (target DNA sequences)^37^. The release of p65/p50 complex from the inhibitor complex requires the activation of classical NF-κB pathway and physiologically linked to the regulation of cellular growth and survival^38–40^. The regulation of cell growth depends on the phosphorylation status of the p65 NF-κB subunit^22^. It had been pointed out that p65 could regulate cell-cycle progression, cell apoptosis and cell growth in various cancer types^41,42^. It is reported that TNF receptor-associated factor 2 (TRAF2) binding to SPHK1 is required for TNF-α-induced nuclear translocation of p65^43^. And SPHK1 is also been identified as a target of miR-506, and knockdown miR-506 could resulted in upregulation of SPHK1 and followed by activation of NF-κB pathway in pancreatic cancer^44^. Combine with our microarray result, we wondered whether NF-κB signaling could act as a downstream effector of POTEE/SPHK1 axis. With this hypothesis, we used p65 inhibitor to verified that gradually suppressed p-p65 could progressively abrogated the effects of cell growth induced by POTEE overexpression and knockdown SPHK1 could also rescue the increase of p-p65 mediated by POTEE overexpression. Collectively, we speculated that POTEE could affect p65 activation by regulating SPHK1 expression.

Nevertheless, since researches on POTEE are extremely limited and the application of POTEE antibody could only be used for western blot and immunohistochemistry, so the precise interaction between POTEE and SPHK1 is still unknown. From our current data, we could only figure out that large amount of POTEE locate in cell nucleus and a small part of it lie in the cytoplasm, and POTEE could activate the mRNA and protein expression of SPHK1. In our hypothesis, POTEE may act as a transcription factor, or directly interact with the upstream signal molecule of SPHK1, or directly interact with SPHK1 for induction of phosphorylation, which might be more complicated and needs further investigation.

In conclusion, our present study sheds light on the oncogenic functions of POTEE in CRC progression. Mechanistically, our results also showed that POTEE might play a critical role in regulating the expression of SPHK1 and followed activation of NF-κB singling, which might act as a novel biomarker and a potential intervention of CRC patients.

## Materials and methods

### Patients and samples

The Institute Research Medical Ethics Committee of Nanfang Hospital (Guangzhou, China) granted approval for this study. Fresh and formalin-fixed tissue samples from patients with CRC were collected from the Department of General Surgery, Nanfang Hospital, affiliated to Southern Medical University. 20 pairs of CRC and non-tumor-adjacent tissue samples from the patients who received curative surgery in the Nanfang Hospital were used for quantitative real-time polymerase chain reaction (qRT-PCR) or western blot analysis and 81 CRC surgical specimens were used for immunohistochemistry (IHC) and prognosis analysis. In addition, a tissue microarray (TMA) contained tumor resections from 83 CRC patients were used to analyze the correlation of protein expression between POTEE and SPHK1.

### Western blot analysis

Cells or tissues were lysed with RIPA buffer (Amresco, America) and total proteins were separated by 10% SDS-PAGE (Amresco, America) and transferred to PVDF membranes. Membranes were incubated with respective primary antibodies overnight at 4 °C and washed three times. Then, these blots were incubated with HRP-conjugated secondary antibody (Cell Signaling Technology #7074, America) for 1 h at room temperature. After washed three times the blots were visualized by enhanced chemiluminescence (FDbio-pico ECL, China). Primary antibodies used for western blot were listed as follows: POTEE (Biorbyt #orb312653, Britain), SPHK1 (Proteintech #10670-1-AP, America), p-SPHK1 (Proteintech #19561-1-AP, America), p65 (Cell Signaling Technology #8402, America), p-p65 (Cell Signaling Technology #3033, America), GAPDH (Cell Signaling Technology #5170, America).

### Quantitative real-time PCR (qRT-PCR)

Total RNAs from cells or tissues were extracted using TRIzol reagent (TaKaRa, Japan) and reverse transcribed into cDNA using a PrimeScript RT-PCR Kit (TaKaRa, Japan) according to the manufacturer’s instructions. Briefly, we analyzed the expressions of mRNA using SYBR™ Premix Ex Taq™ (TaKaRa, Japan) under the LightCycler 96 Detection System (Roche) and GAPDH was used for normalization. Primer sequences for quantitative real-time PCR were listed in Table 2.

**Table 2:** Primer sequences for qRT-PCR in this study

GAPDH-FP	3′-TGACTTCAACAGCGACACCCA-5′	GAPDH-RP	3′-CACCCTGTTGCTGTAGCCAAA-5′
POTEE-FP	3′-CTGCACTACGCTATCTATAA-5′	POTEE-RP	3′-TCAAGTAGAAGGCTGACTAT-5′
TGFA-FP	3′-AGGTCCGAAAACACTGTGAGT-5′	TGFA-RP	3′-AGCAAGCGGTTCTTCCCTTC-5′
DDR1-FP	3′-CCGACTGGTTCGCTTCTACC-5′	DDR1-RP	3′-CGGTGTAAGACAGGAGTCCATC-5′
GNAI1-FP	3′-CTGCGCTGGATGCTTGATTTT-5′	GNAI1-RP	3′-GATTCACCAGCACCTGGCAA-5′
SPHK1-FP	3′-GCTGCGAAGTTGAGCGAAAA-5′	SPHK1-RP	3′-GGCTGGACCCAGTCGGCG-5′
BMP2-FP	3′-ACTACCAGAAACGAGTGGGAA-5′	BMP2-RP	3′-GCATCTGTTCTCGGAAAACCT-5′
BMPR2-FP	3′-GACAGGAGACCGTAAACAAGG-5′	BMPR2-RP	3′-CCATATCGACCTCGGCCAATC-5′
CSNK2A2-FP	3′-AAAAGCTGCGACTGATAGATTGG-5′	CSNK2A2 -RP	3′-GAGGCTACACGAACATTGTACTC-5′
RND2-FP	3′-AAGATCGTGGTGGTGGGAG-5′	RND2-RP	3′-CATAACTCCCGGGATAGGCG-5′
IRS2-FP	3′-GGCCACCATCGTGAAAGAGTG-5′	IRS2-RP	3′-GTGACCTTGCCTTGTTGGTG-5′
DIRAS3-FP	3′-AGCTTATTCCAACAGATGCCA-5′	DIRAS3-RP	3′-AACCAACTGGACACCCCAAG-5′
PLCB4-FP	3′-ACATGCCTCAAGAAACACTGGA-5′	PLCB4-RP	3′-GCTGTGGGCTCAATTTCATCA-5′
BMP4-FP	3′-ATGATTCCTGGTAACCGAATGC-5′	BMP4-RP	3′-CCCCGTCTCAGGTATCAAACT-5′
BCL10-FP	3′-GCAGTTGTGAACCTTTTCCAGA-5′	BCL10 -RP	3′-TGGATGCCCTCAGTTTTTCAG-5′
ROCK1-FP	3′-GGTCTAAATAGGTCGTCGTACAA-5′	ROCK1-RP	3′-TCCGTACCATGCTACCATATGT-5′

### Immunohistochemistry (IHC)

Slides were stained using the MaxVision TM^2^ Kit (MXB® Biotechnologies, China) according to the manufacturer’s instructions. At least three individual fields (20×) were chosen to estimate the score of each slide. Immunostaining intensity was divided into 4 grades: 0, negative; 1, weak; 2, moderate; 3, strong. As well as the proportion of staining-positive cells: 0, < 5%; 1, 6–25%; 2, 26–50%; 3, 51–75%; 4, >75%. Each slide was scored by the cross product of the value of proportion of staining-positive cells and the value of immunostaining intensity. Two independent and experienced pathologists were invited to assess and confirm the results. Primary antibodies were POTEE (Biorbyt #orb312653, Britain), SPHK1 (Proteintech #10670-1-AP, America), p-p65 (Cell Signaling Technology #3033, America), Ki67 (Cell Signaling Technology #9449, America).

### Cell culture and stable cell lines construction

The CRC cell lines RKO, SW480, and CACO2 were purchased from the American Type Culture Collection and maintained at 37°C in an atmosphere containing 5% CO_2_. The cells were cultured in 1640 medium supplemented with 10% fetal bovine serum (Gbico, America). Lentiviral vectors were designed and constructed by GENECHEM Biotech at Shanghai, China (http://genechem.bioon.com.cn/). Flag-tagged POTEE-overexpressed vectors (LV-POTEE) and control vectors (LV-NC) were transfected into RKO and CACO2 cells, while POTEE shRNA (shPOTEE) and control short hairpin RNA (shNC) were transfected into RKO and SW480 cells to generate POTEE-knockdown cells. Moreover, SPHK1 shRNA (shSPHK1) and control short hairpin RNA (shRNA) were transfected into LV-NC or LV-POTEE cells above mentioned. Transfection procedures were performed according to the manufacturer’s instruction.

### MTT and colony formation assay

For MTT assays, stably transfected cells (1000 cells per well) were cultivated on 96-well plates and cell proliferation were detected for 6 days with MTT (Beyotime, China) at 570 nm. For the colony formation assays, cells (500 cells per well) were cultivated in six-well plates and maintained with 1640 medium containing 10% FCS at 5% CO2, 37 °C for 8–12 days. At the end of experiments, colonies formed were washed with phosphate buffer (PBS), fixed in methanol and stained with 0.1% crystal violet. Colonies containing more than 50 cells for each well were counted (in triplicate).

### Cell migration and invasion assay

For scratch test, cells was cultural about 70–80% confluence in six-well plate. Scratches were made by 20 µl pipette tip. Then the cells were cultural under serum-starved condition. Images were taken at 0 h, 24 h, and 48 h to evaluate the wound-healing. Eight micrometer pore insets transwell chambers were used for migration and invasion assay. For migration assay, 1 × 10^5^ cells were plated on uncoated insets with serum-starved condition. For invasion assay, uncoated insets were filled with 100 µl 1:20 diluted matrigel and 1 × 10^6^ cells were plated on the above insets with serum-starved condition. The lower chamber contained 600 μl culture medium containing 20% FBS. After 36–48 h incubation, invaded cells were fixed with methanol and stained with 5% crystal violet.

### In vivo subcutaneous xenograft models

All nude mice were purchased from Laboratory Animal Center of Southern Medical University and were humanly treated under the guidelines of the Treatment Committee of Southern Medical University. Stable transfection cells with POTEE knockdown and control cells (5 × 10^7^) were transplanted subcutaneously into the bilateral flanks of 3–4 weeks old nude mice. Tumor diameters were measured every 3 days, and the formula Tumor Volume (mm^3^) = (length × width^2^)/2 was used to evaluated the tumor volumes. Animals were sacrificed 27 days after injection and the tumor tissues were fixed in formalin for IHC analysis.

### Flow cytometry

For cell-cycle analysis, cells under detection were collected and then fixed in 70% ethanol overnight. Next day, the cells were washed with PBS and incubated with RNase A solution and PI staining. The detailed procedures were performed according to the manufacturer’s instructions (KeyGEN BioTECH, China). For apoptosis assay, stable transfected cells under detection were collected after 5-Fu treatment for 36–48 h. Cells were then collected and apoptosis was then detected with Annexin V-FITC/PI cell apoptosis detection kit (KeyGEN BioTECH, China).

### Microarray analysis

POTEE-overexpressed RKO cells and negative control cells were used for gene expression profiling. Intactness of the samples was assessed by the Agilent 2100 bioanalyzer (Agilent, America). Identification of differentially expressed genes was performed by Shanghai Genechem Co., LTD (Shanghai, China). Microarray analysis of mRNA profiles using Gene Matrix platforms (http://gcloud.taogene.com) based on IPA analysis.

### Statistical analysis

The results were shown as the mean ± SD and *P* < 0.05 was considered to be statistically significant. The differences in survival rates were assessed by Kaplan–Meier analysis. Pearson χ2 test was used to analyze the correlation between the expression of POTEE and SPHK1. All of the statistical analyses were carried out with SPSS software (version 16.0) or GraphPad Prism (version 6.0).

## Supplementary information

Supplementary figures information

Supplementary Figure 1

Supplementary Figure 2

Supplementary Figure 3

Supplementary Figure 4

Supplementary Figure 5
