# Supplementary material for: POTEE drives colorectal cancer development via regulating SPHK1/p65 signaling
Source: Cell Death Dis. 2019 Nov 13;10(11):863. doi: 10.1038/s41419-019-2046-7 (PMC6853991; doi:10.1038/s41419-019-2046-7)
Supplement: Supplementary file 1 — Supplementary figures information [file 41419_2019_2046_MOESM1_ESM.docx]

**Supplemental Figure 1：POTEE was primarily located at the nucleus.**

**a**. Immunohistochemistry (IHC) of POTEE in CRC sample.

**b**. Western blot was performed to show nuclear and cytoplasmic protein to identify the location of endogenous POTEE protein.

**Supplemental Figure 2：POTEE plays oncogenic roles in CRC cells.**

**a.** Scratch test in indicated cell lines with different POTEE expression. Statistics were measured by Student t, ^*^*P* < .05, ^**^*P* < .01, ^***^*P* < .001.

**b.** Migration assay in indicated cell lines with different POTEE expression. Statistics were measured by Student t, ^*^*P* < .05, ^**^*P* < .01, ^***^*P* < .001.

**Supplemental Figure 3：Heat map of genes expression in RKO with POTEE overexpression and control cells based on microarray analysis.**

**Supplemental Figure 4：POTEE facilitates proliferation through up-regulating SPHK1.**

**a, b**. qRT-PCR (**a**) and western blot (**b**) were performed to examine POTEE and SPHK1 endogenous expression in 11 colon cancer cell lines.

**c**. SPHK1 knockdown was verified in control and POTEE-overexpressed cell lines.

**d, e**. MTT (**d**) and colony formation assays (**e**) in cells with different POTEE and SPHK1 expression. Statistics were measured by 2-way ANOVA or Student t, ^*^*P* < .05, ^**^*P* < .01, ^***^*P* < .001.

**Supplemental Figure 5：p65 plays as a functional downstream molecular of POTEE/SPHK1 axis.**

**a**. Western blot analysis detected p53 protein in indicated cell lines with different POTEE expression.

**b**. Western blot analysis in indicated cell lines with EVP4593 (50 nM, 48h) or DMSO treatment.

**c, d**. MTT (**c**) and colony formation assays (**d**) in cells with EVP4593 or DMSO treatment. Statistics were measured by 2-way ANOVA or Student t, ^*^*P* < .05, ^**^*P* < .01, ^***^*P* < .001.

**e**. p65 and p-p65 expression in cell lines with POTEE overexpression and SPHK1 knockdown.
