# Supplementary figures and images for: POTEE drives colorectal cancer development via regulating SPHK1/p65 signaling
Source: Cell Death Dis. 2019 Nov 13;10(11):863. doi: 10.1038/s41419-019-2046-7 (PMC6853991; doi:10.1038/s41419-019-2046-7)

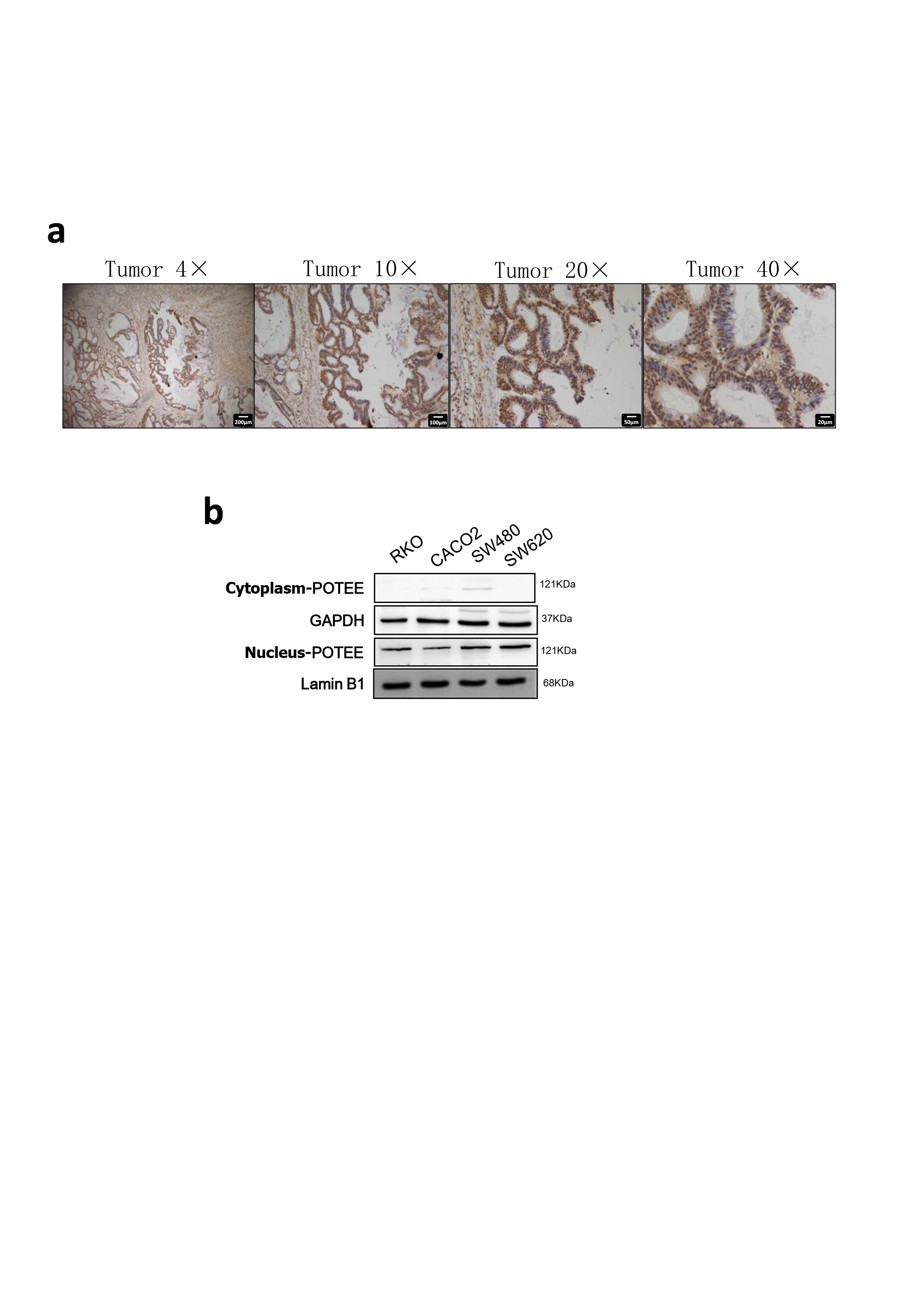

Supplement: Supplementary file 2 — Supplementary Figure 1 [file 41419_2019_2046_MOESM2_ESM.jpg]

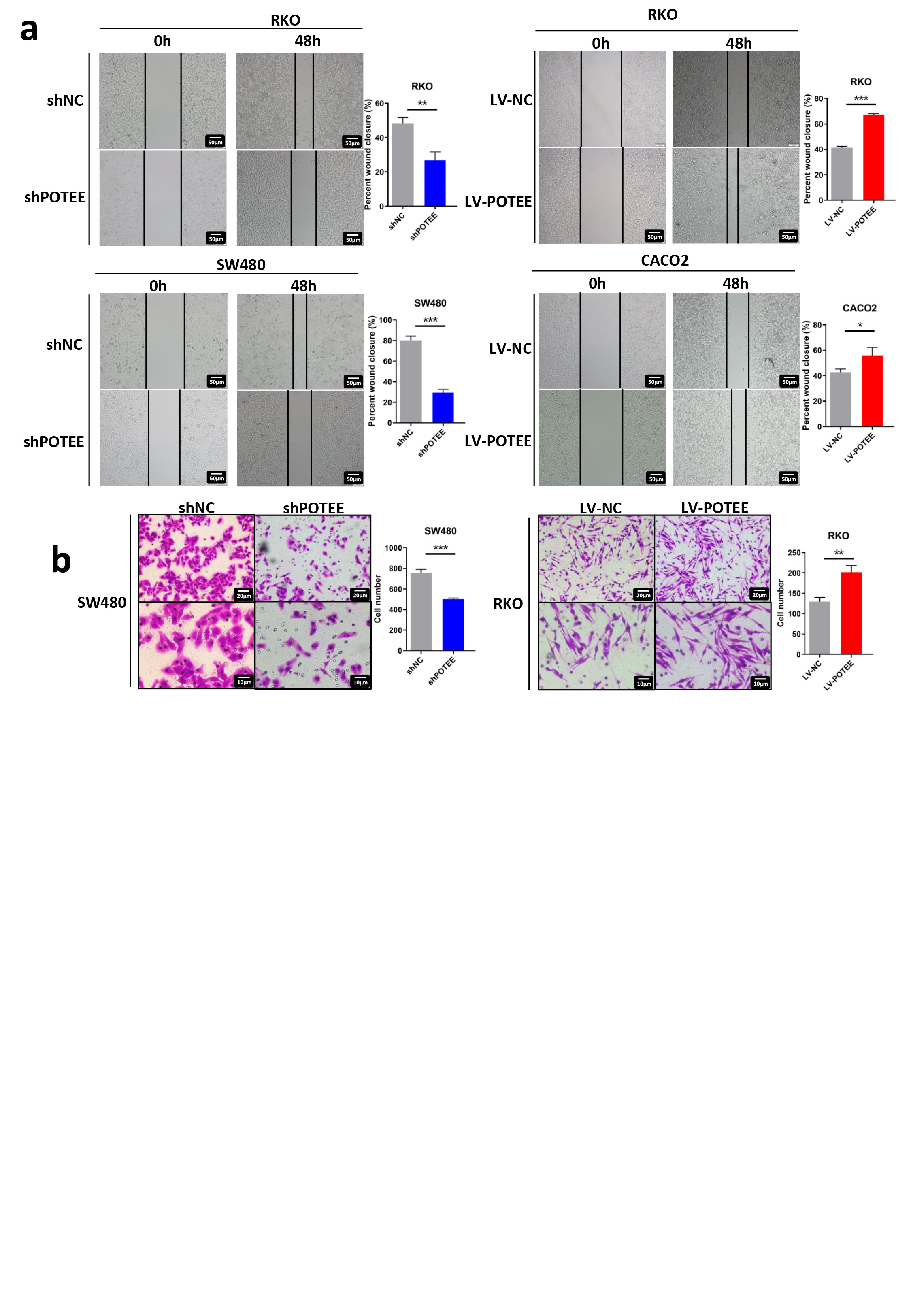

Supplement: Supplementary file 3 — Supplementary Figure 2 [file 41419_2019_2046_MOESM3_ESM.jpg]

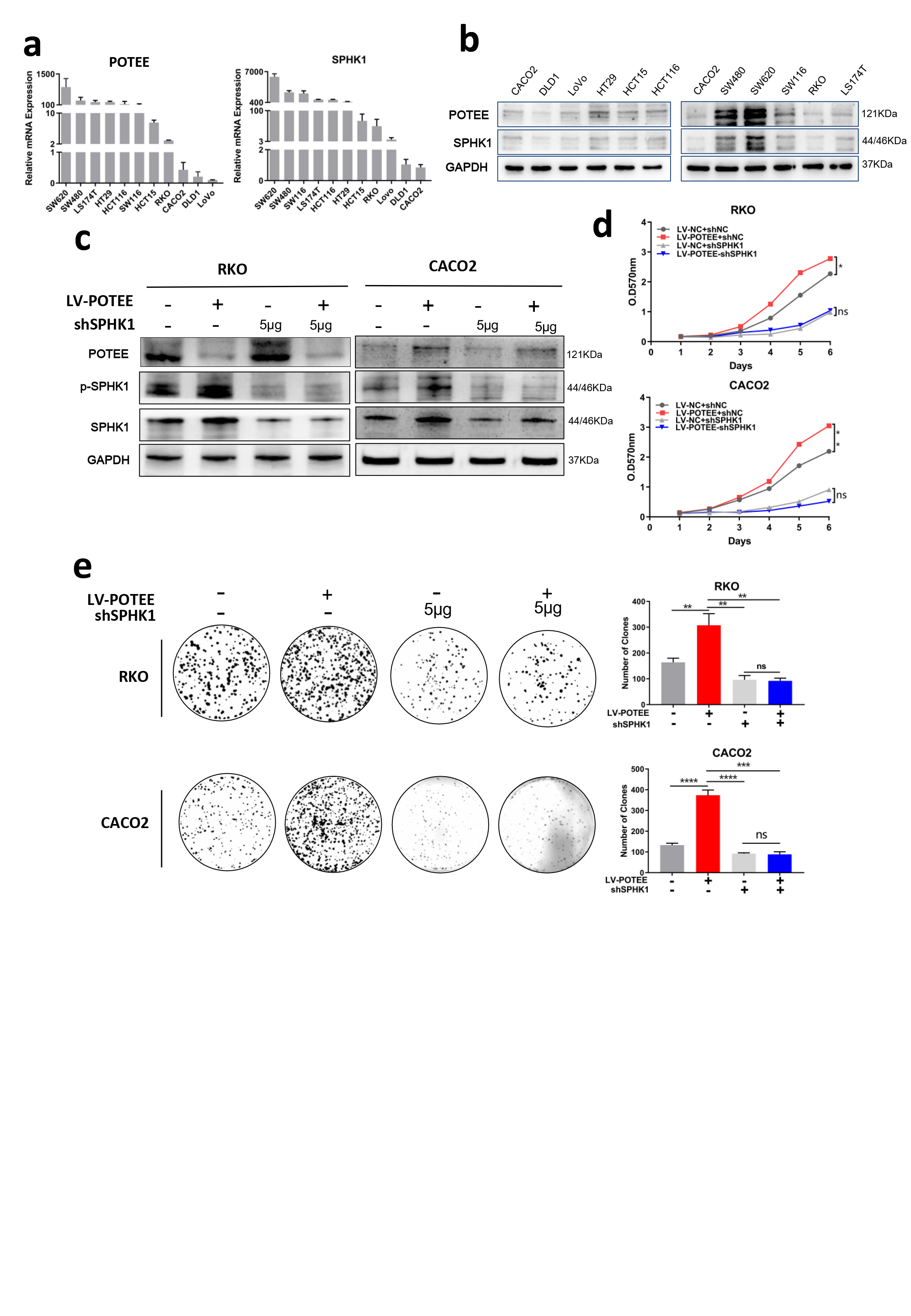

Supplement: Supplementary file 5 — Supplementary Figure 4 [file 41419_2019_2046_MOESM5_ESM.jpg]

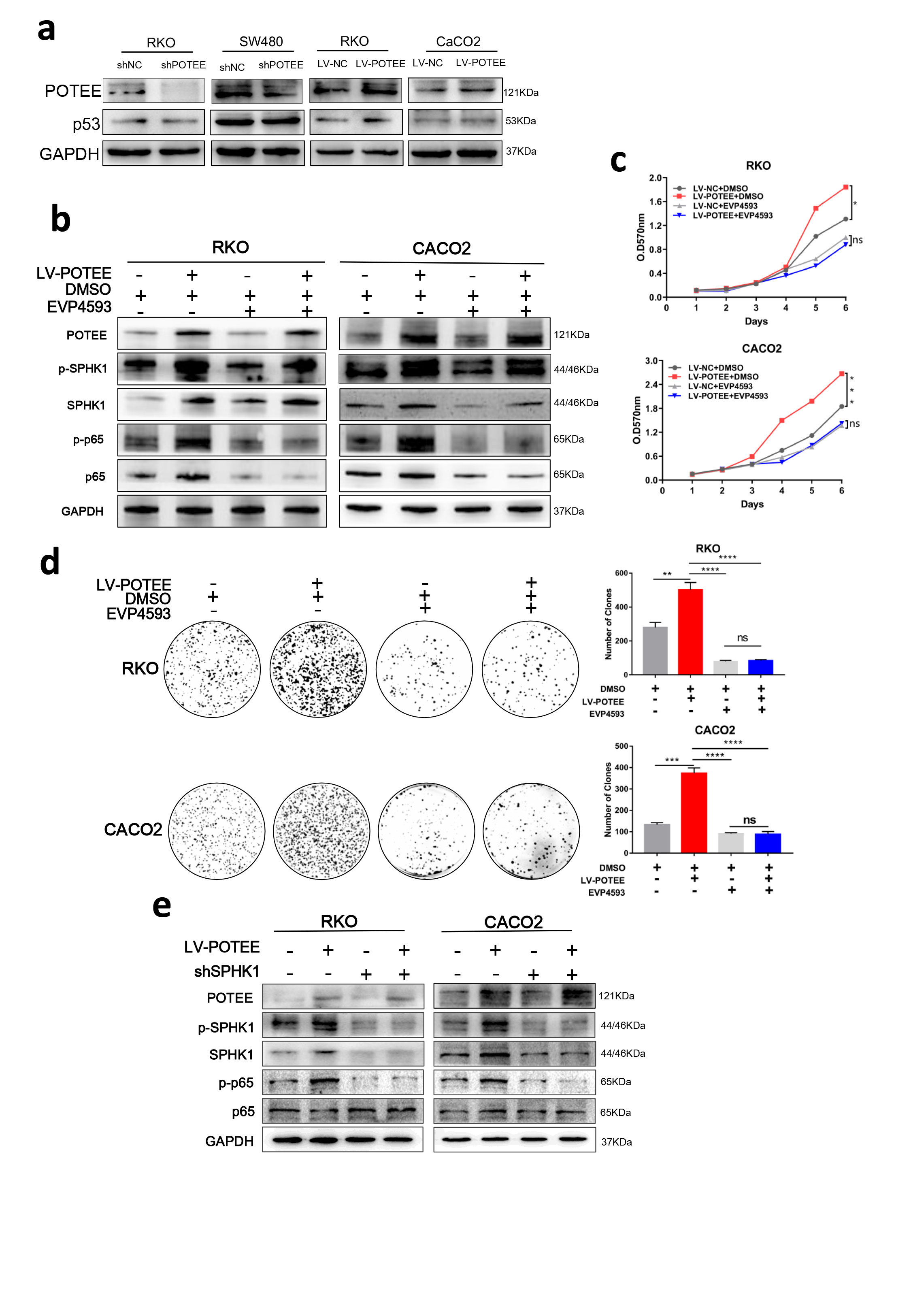

Supplement: Supplementary file 6 — Supplementary Figure 5 [file 41419_2019_2046_MOESM6_ESM.jpg]
